# The clinical potential of optogenetic interrogation of pathogenesis

**DOI:** 10.1002/ctm2.1243

**Published:** 2023-05-02

**Authors:** Tianyu Terry Gao, Teak‐Jung Oh, Kritika Mehta, Yu‐En Andrew Huang, Tyler Camp, Huaxun Fan, Jeong Won Han, Collin Michael Barnes, Kai Zhang

**Affiliations:** ^1^ University of Illinois at Urbana‐Champaign Department of Biochemistry Urbana Illinois USA; ^2^ University of Illinois at Urbana‐Champaign Center for Biophysics and Quantitative Biology Urbana Illinois USA; ^3^ Cancer Center at Illinois University of Illinois at Urbana‐Champaign Urbana Illinois USA

**Keywords:** channelrhodopsin, non‐opsin‐based, opsin‐based, optogenetics, photoactivatable proteins, protein‐protein interaction

## Abstract

**Background:**

Opsin‐based optogenetics has emerged as a powerful biomedical tool using light to control protein conformation. Such capacity has been initially demonstrated to control ion flow across the cell membrane, enabling precise control of action potential in excitable cells such as neurons or muscle cells. Further advancement in optogenetics incorporates a greater variety of photoactivatable proteins and results in flexible control of biological processes, such as gene expression and signal transduction, with commonly employed light sources such as LEDs or lasers in optical microscopy. Blessed by the precise genetic targeting specificity and superior spatiotemporal resolution, optogenetics offers new biological insights into physiological and pathological mechanisms underlying health and diseases. Recently, its clinical potential has started to be capitalized, particularly for blindness treatment, due to the convenient light delivery into the eye.

**Aims and methods:**

This work summarizes the progress of current clinical trials and provides a brief overview of basic structures and photophysics of commonly used photoactivable proteins. We highlight recent achievements such as optogenetic control of the chimeric antigen receptor, CRISPR‐Cas system, gene expression, and organelle dynamics. We discuss conceptual innovation and technical challenges faced by current optogenetic research.

**Conclusion:**

In doing so, we provide a framework that showcases ever‐growing applications of optogenetics in biomedical research and may inform novel precise medicine strategies based on this enabling technology.

## Graphical abstract

1. Opsin‐based and opsin‐free optogenetics employs light to modulate membrane conductance and protein‐protein interaction.
2. Clinical trials focus on treating blindness due to the easy delivery of light.
3. Protein engineering improves the photophysics and biocompatibility of photoactivatable proteins.
4. Opsin‐free optogenetics expands clinical applications such as the chimeric antigen receptor (CAR) and clustered regularly interspaced short palindromic repeats (CRISPR)‐based genomic engineering and transcriptional regulation.

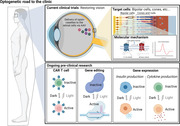

## 1 BACKGROUND

Insights into pathogenesis are crucial for disease prevention, diagnosis, management, and treatment. Disease etiology and progression often involve disruptive gene modification, defective intracellular and intercellular cell signaling, and dysregulated cell‐environment communications, which could intervene with each other. For example, gain‐ or loss‐of‐function gene mutation could result in misregulated protein‐protein interaction (PPI) and modified intra‐ or intercellular signaling. Mechanistic understanding and rescue of pathogenesis require strategies to visualize and modify molecular activity with cell‐type specificity and spatiotemporal accuracy to avoid systemic changes that may distort systemic physiology. This demand has inspired the development of new model systems (e.g., organoid, organ‐on‐a‐chip), molecular probes (organic or genetic), labeling strategies (e.g., those based on unnatural amino acids or click chemistry), imaging modality (super‐resolution and live‐cell imaging) and sequencing strategies (genomic, transcriptomic, epigenomics and metabolomic) to enable precise *visualization* of biological systems. On the other hand, spatiotemporal *modulation* of molecular activity and signal transduction remains challenging.

The emerging optogenetics offers unique features to empower new modes to control molecular activity and cell signaling. Optogenetics uses a suite of photoactivatable proteins, which undergo conformational changes upon exposure to light at specific wavelengths, to interrogate molecular activity and complex intracellular signaling networks.^1^, ^2^, ^3^, ^4^, ^5^ Blessed by the capacity to draw the causal link between neural circuits and behaviour, optogenetic technology has been awarded as the Methods of the Year 2010.^6^ Shortly after the report of light‐gated opsin‐based neuronal firing control,^7^ optogenetics was successfully used to control various biological events in cells (see recent reviews^8^, ^9^, ^10^, ^11^, ^12^, ^13^, ^14^, ^15^) and multicellular organisms.^16^

Optogenetics's spatial and temporal accuracy provides insights into translational research,^17^ such as in pain management,^18^ strokes,^19^ epilepsy,^20^ behavior,^21^ heart diseases,^22^, ^23^, ^24^ motor functions,^25^ memory^26^ and psychiatric diseases, including depression, anxiety, addiction, schizophrenia, and autism, to name a few.^27^ However, technical challenges, such as light delivery, transgene delivery, sensitivity, and toxicity assessment, should be addressed before realizing the full clinical potential of optogenetics.^28^, ^29^, ^30^ Here, we introduce commonly used opsin‐based and opsin‐free optogenetic tools, followed by a summary of ongoing clinical trials, primarily of opsin‐based optogenetics. We then expand the landscape of biological applications by showcasing opsin‐free optogenetics. A section is dedicated to discussing key challenges in translating optogenetics to clinical research, concluded by a perspective ‘wishlist’ for next‐generation optogenetics. We hope this work can stimulate more discussions from researchers in both fundamental and clinical research and maximally leverage features of optogenetics in clinical applications.

## 2 PRINCIPLES OF OPSIN‐BASED OPTOGENETICS

### 2.1 Structure and photophysics of microbial opsin

**FIGURE 1:**
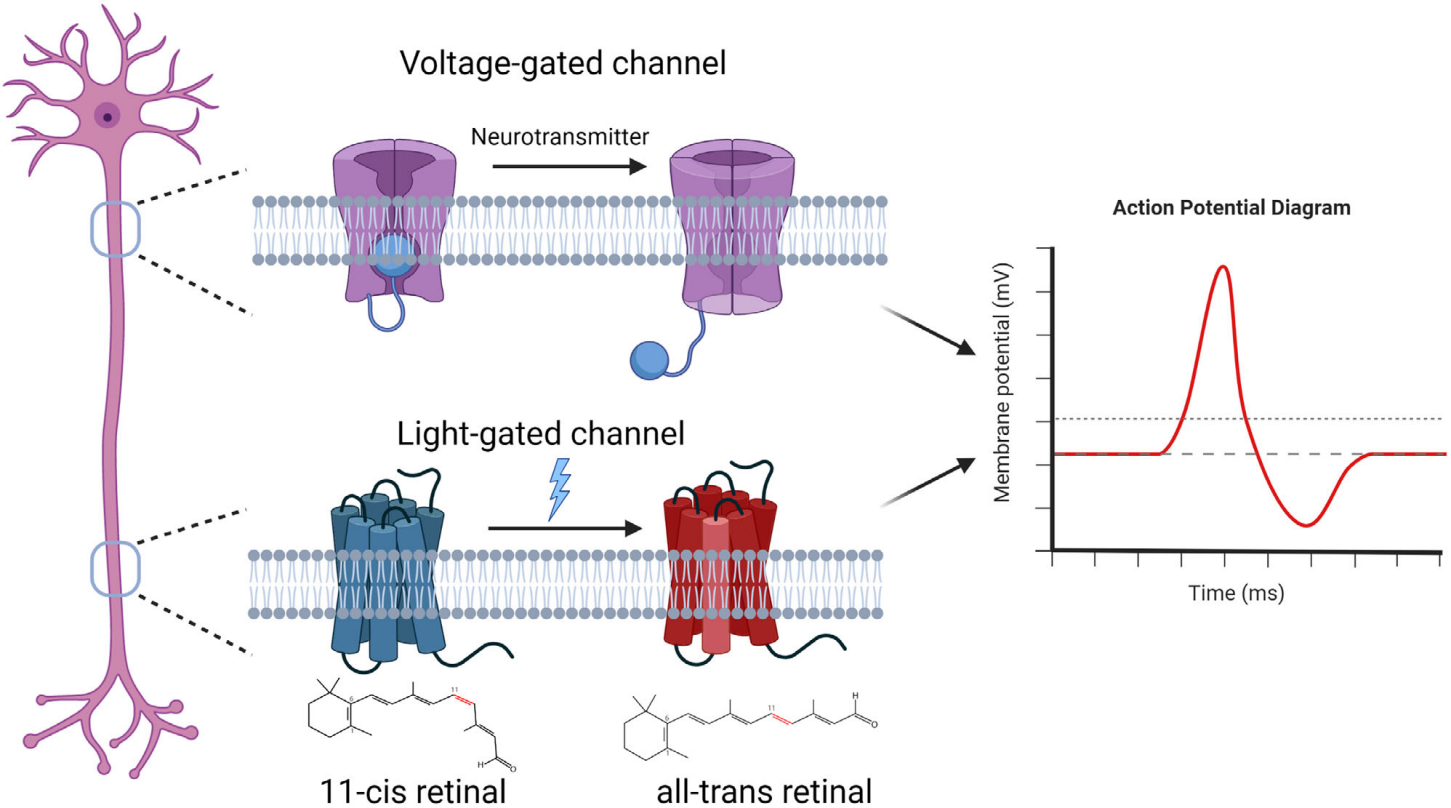
Comparison between ligand and light‐gated ion channel for action potential induction. In excitable cells such as neurons, binding neurotransmitters to voltage‐gated channels induces a conformational change of the channel, allows ion flow across the membrane to generate action potentials. Channelrhodopsin and variants utilize light‐induced cis‐trans conformational changes of its retinal cofactor to tune channel conductance and generate action potentials.

Optogenetics uses light to control protein conformation and empowers new ways to control molecular activity in live cells. Opsin‐based optogenetics enabled accurate control of ion flux across the cell membrane through light‐sensitive channels or pumps.^7^, ^31^, ^32^, ^33^ Successful implementation of opsin‐based optogenetics requires (1) microbial opsins that transport ions upon exposure to light, (2) a strategy to express opsins in specific target cells, and (3) precise delivery of light to the target cells.^34^ Channelrhodopsin‐2 (ChR2), the opsin derived from the green freshwater algae *Chlamydomonas reinhardtii*, is a seven‐pass transmembrane protein that belongs to microbial opsins. ChR2 binds a retinal cofactor that undergoes cis‐trans conformational changes when absorbing blue light. Such a conformational change renders an increased cations influx (Na^+^ and Ca^2+^) through ChR2 and stimulates action potential in hosting excitable cells (e.g., neurons or muscles)^35^, ^36^ (Figure 1). ChR variants such as halorhodopsins (HRs) pump chloride ions into the cells upon light illumination, leading to optical induction of hyperpolarization and neuronal inhibition.^37^ Genetic ChR expression can be virally driven (lentivirus or adeno‐associated virus) in cells or animals. Cre‐dependent ChR expression transgenic mice have also been developed (e.g., strain Ai32, the Jackson Laboratory) to facilitate the production of animals bearing the tissue‐specific expression of ChR2.

### 2.2 The clinical accomplishment of opsin‐based optogenetics

Current clinical trials of optogenetics mainly focus on treating blindness due to the easy delivery of light through the eyes. We discuss six records of clinical trials, five active and one completed, which target retinal diseases. Key information about each clinical trial is listed in Table 1.

**TABLE 1:** Opsin‐based optogenetics in current clinical trials for retinal diseases.

Trial #	Target	Stage	Intervention	Participant (#; label)	Start‐end yr	Status	Sponsor
**NCT02556736**	Advanced RP	Phase I/IIa	Intravitreal injection ChR2‐encoding AAV	14; Open‐label	2015–2024	Active, not recruiting	AbbVie
**NCT04919473**	Advanced RP	Phase I/IIa	MCO‐encoding AAV2	11; Non‐randomized, Open‐label	2019–2020	Completed	Nanoscope Therapeutics Inc.
**NCT04945772**	RP	Phase IIb	MCO‐encoding AAV2	27; Randomized, Double‐masked, Sham‐controlled	2021–2024	Active, not recruiting	Nanoscope Therapeutics Inc.
**NCT05417126**	Stargardt Disease	Phase IIa	MCO‐encoding AAV2	6; Open‐label	2022–2023	Recruiting	Nanoscope Therapeutics Inc.
**NCT03326336**	Non‐syndromic RP	Phase I/IIa	ChrimsonR‐tdTomato‐encoding AAV2.7	15; Non‐randomized, Open‐label	2018–2025	Recruiting	GenSight Biologics
**NCT05294978**	IRDs	N/A	Diagnostic Test: OCT	1000; Case‐controlRetrospective	2021–2023	Recruiting	University Hospital, Basel, Switzerland

Abbreviations: IRD, inherited retinal dystrophies; MCO, multi‐characteristic opsin; RP, retinitis pigmentosa.

Clinical trial name: NCT02556736: RST‐001 Phase I/II Trial for Advanced Retinitis Pigmentosa; NCT04919473: Dose‐Escalation Study to Evaluate the Safety and Tolerability of Intravitreal vMCO‐I in Patients With Advanced Retinitis Pigmentosa; NCT04945772: Efficacy and Safety of vMCO‐010 Optogenetic Therapy in Adults With Retinitis Pigmentosa; NCT05417126: Safety and Effects of a Single Intravitreal Injection of vMCO‐010 Optogenetic Therapy in Subjects With Stargardt Disease; NCT03326336: Dose‐escalation Study to Evaluate the Safety and Tolerability of GS030 in Subjects With Retinitis Pigmentosa; NCT05294978: EyeConic: Qualification for Cone‐Optogenetics

The fundamental idea of using light to treat retinal diseases is to revamp defective visionary neurons with light sensitivity. Retinitis pigmentosa (RP) is a genetic disease that affects the retina and causes the breakdown of photoreceptors, affecting the peripheral vision first and eventually spreading to the central vision.^38^ Stargardt's disease was a genetic disease with fatty material build up on the retina's central region (macula), resulting in the loss of central vision and light sensitivity.^39^ AbbVie sponsored the earliest phase I/II clinical trials to determine the dose‐dependent safety and tolerability of single‐dose intravitreal RSO‐001 to advanced RP patients (**NCT02556736**). Based on the results updated on 6 October 2022, of the 14 participants, the treatment resulted in 0% all‐cause mortality or serious adverse events. Nine participants experienced grade 3, non‐life threatening but medically significant side effects, including mild eye discharge, irritation, pain, and infection. A dose‐escalation trial was carried out to evaluate the safety and tolerability of adeno‐associated virus (AAV) as the transgene vehicle. This completed phase I/IIa clinical trial by Nanoscope Therapeutics (**NCT04919473**) evaluated patient tolerance to vMCO‐I, a serotype 2 AAV carrying a multi‐characteristic opsin (MCO) gene expression cassette. Eleven patients with advanced RP received intravitreal vMCO‐I at high (3.5E11 viral genome per eye) or low (1.75E11 viral genome per eye) doses. vMCO‐I infects and expresses polychromatic opsin, which can be activated by ambient light, in patients’ bipolar cells. All subjects had objective and subjective improvement in functional vision, including shape discrimination accuracy improved to greater than 90% in all subjects compared to baseline. Following the phase I/IIa trial (**NCT04919473**), Nanoscope Therapeutics started a phase IIb trial (**NCT04945772**) that addresses the efficacy and systemic adverse effects at a lower dose (dropping from 3.5E11‐1.75E11 to 1.2E11‐0.9E11). The same company has another ongoing phase IIa clinical trial that assesses the safety and efficacy of vMCO‐010 for Stargardt's disease (**NCT05417126**).

Recently, GenSight Biologics reported promising efficacy results in the **NCT03326336** trial, which showed partial visual function recovery in a blind RP patient. The patient received an intraocular injection of an AAV vector (5E10 viral genome per eye) encoding ChrimsonR, a red light (590 nm) responsive opsin. The red light was delivered through an engineered goggle to stimulate human retinal ganglion cells. During 84 weeks of treatment, the patient perceived, located, counted, and touched different objects. In addition, the patient's occipital neural activity assessment was also successfully correlated with functional visual recovery.

Besides bipolar cells, University Hospital, Basel, Switzerland, initiated a clinical trial (**NCT05294978**) in 2021 targeting cone cells to restore visions in patients with inherited retinal dystrophies. Cone cells are crucial in color vision, daytime activity, and faster bright light flash recovery. A niche is that light‐sensitive cones remain alive but dormant before the disease progresses into the late stage, posing an excellent time window to re‐sensitize them through optogenetics. However, the population of patients in the dormant stage is unknown. The primary aim of this study is to identify eligible patients worldwide (USA, China, Germany, Hungary, Italy, Switzerland, and the UK) through a multicenter ocular imaging study (EyeConic Study) for cone‐optogenetics treatment. Currently, there are 1000 patients enrolled, and their eligibility will be determined via macular optical coherence tomography, which captures the pathogenic state of the retina, as well as deeper eye structure and vasculature, non‐invasively.

## 3 OPSIN‐FREE OPTOGENETICS EXPANDS THE SCOPE OF OPTICALLY CONTROLLED BIOLOGICAL PROCESSES

### 3.1 Structure and photophysics of opsin‐free photoactivatable proteins

Opsin‐free optogenetics shares common prerequisites, such as photoactivatable proteins, transgene expression, and light delivery, as opsin‐based technology. In contrast to fluorescent proteins, optical probes for localizing proteins, photoactivatable proteins serve as actuators to tune target molecules’ activity. Light stimulation of the fluorophore of these photoactivable proteins modifies PPIs, which expands the mode of action to modulate molecular activity and the scope of biological processes.

**FIGURE 2:**
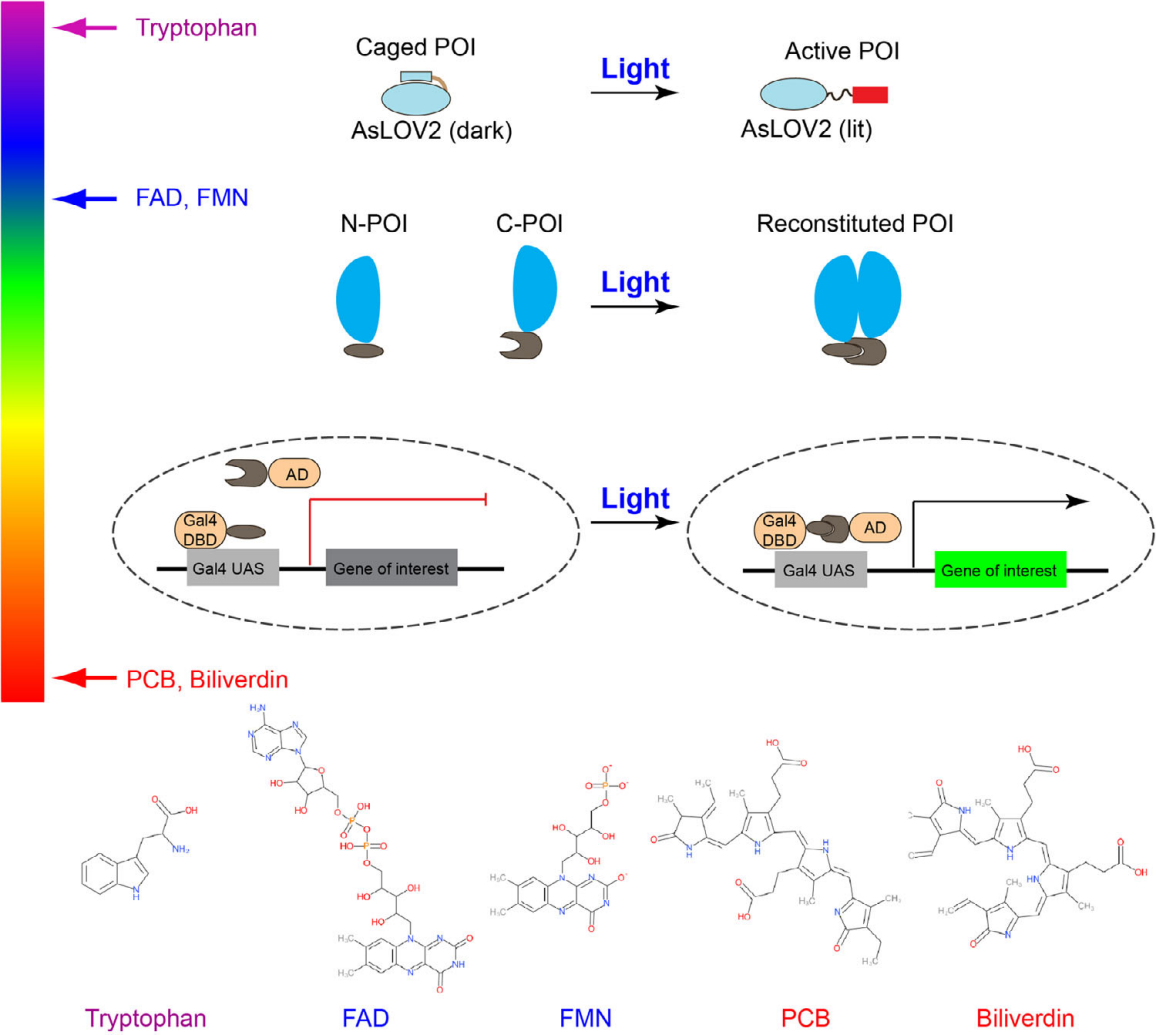
Commonly used modes of action of opsin‐free photoactivable proteins and light‐sensitive chromophores. Modes of action include photo‐uncaging and photo‐inducible recombination of dimerizer for reconstituting split protein or transcriptional control. More details on the excitation wavelength, chromophore, and protein structure can be found in Table 2.

A photoactivatable protein's core components are the photosensory and effector domains. Photosensory domains contain chromophores, excitation of which converts energy stored in photons to the chemical potential that changes the conformation of host molecules. Chromophore excitation results in conformational changes that lead to protein association, dissociation, and uncaging or allosteric effects in host photoactivatable proteins. For example, blue light excitation of flavin mononucleotide (FMN) cofactor in *Avena sativa* light, oxygen, or voltage domain (AsLOV) results in its formation of an adduct with the proximal cysteine residue, which induces conformational changes in LOV. Cryptochrome uses flavin adenine dinucleotide (FAD) to mediate its light‐regulated homo‐oligomerization or heterodimerization. Phytochromes use phycocyanobilin (PCB) or biliverdin, red and far red light‐sensitive chromophores, for conformational modulation. To date, crystal structures and light‐sensitive chromophores of various photoactivatable proteins have been resolved (Figure 2, Table 2). The identified modes of action help the structure‐guided design of innovative optogenetic systems. We will use recent studies to showcase each mode of action, such as photo‐uncaging and association.

**TABLE 2:** Structure and chromophore for selected opsin‐free photoactivatable proteins.

University of Il Opsin‐free photoactivable protein	Excitation wavelength	Chromophore	PDB ID	Reference
UVR8/COP1	300 nm	Tryptophan ‘pyramid’	7VGG	^97^
UVR8/UVR8	300 nm	Tryptophan ‘pyramid’	4D9S	^98^
CRY2/CIB1	450 nm	FAD	7X0Y (CRY2 tetramer + CIB1 fragment) 6K8I (CRY2)	^99^
CRY2/CRY2	450 nm	FAD	6K8I	^100^
AsLOV2	450 nm	FMN	2V1A (dark) 2V1B (light)	^101^
iLID	450 nm	FMN	4WF0	^102^
RsLOV	450 nm	FMN	4HJ4	^65^
vfAuLOV	450 nm	FMN	3UE6	^103^
EL222	450 nm	FMN	3P7N	^104^
VVD	450 nm	FMN	3RH8	^105^
Dronpa145K/N	500 nm	Cys62‐Tyr63‐Gly64 (CYG)	2Z1O (only Dronpa145K)	^106^
Dronpa145N	500 nm	Cys62‐Tyr63‐Gly64 (CYG)	2POX	^107^
pdDronpa1	500 nm	Cys62‐Tyr63‐Gly64 (CYG)	6D39	^66^
TtCBD	545 nm	AdoCbl, MetCbl or CNCbl	No PDB entry	^108^
PhyA/FHY1	660 nm	PCB	No PDB entry	^109^
PhyB/PIF3 & PhyB/PIF6	660 nm	PCB	4OURz	^110^
CPH1	660 nm	PCB	2VEA	^111^
BphS	660 nm	Biliverdin	No PDB entry	^112^

Abbreviations: FAD, flavin adenine dinucleotide; FMN, flavin mononucleotide; LOV, light, oxygen, or voltage; PCB, phycocyanobilin; PDB, protein data bank; VVD, vivid.

## 4 CONCEPTUAL INNOVATION OF OPTOGENETICS IN PRECLINICAL RESEARCH

Optogenetics's conceptual innovation leverages physics (the ‘opto’ part) and biology (the ‘genetics’ part), providing new modalities, such as spatiotemporal accuracy, target specificity, and tunable kinetics, when integrated into other therapies (Mathony et al., 2020; Tan et al., 2017) and drug discovery processes (Kiełbus et al., 2018). Besides benefiting fundamental research, these features continue pushing optogenetics into translational, preclinical, and clinical research (Bansal et al., 2022) against diseases such as cancer (Malogolovkin et al., 2022), diabetes (Chen et al., 2022) or other multitudes of diseases (Ye and Fussenegger, 2019).

### 4.1 Spatiotemporal accuracy

**FIGURE 3:**
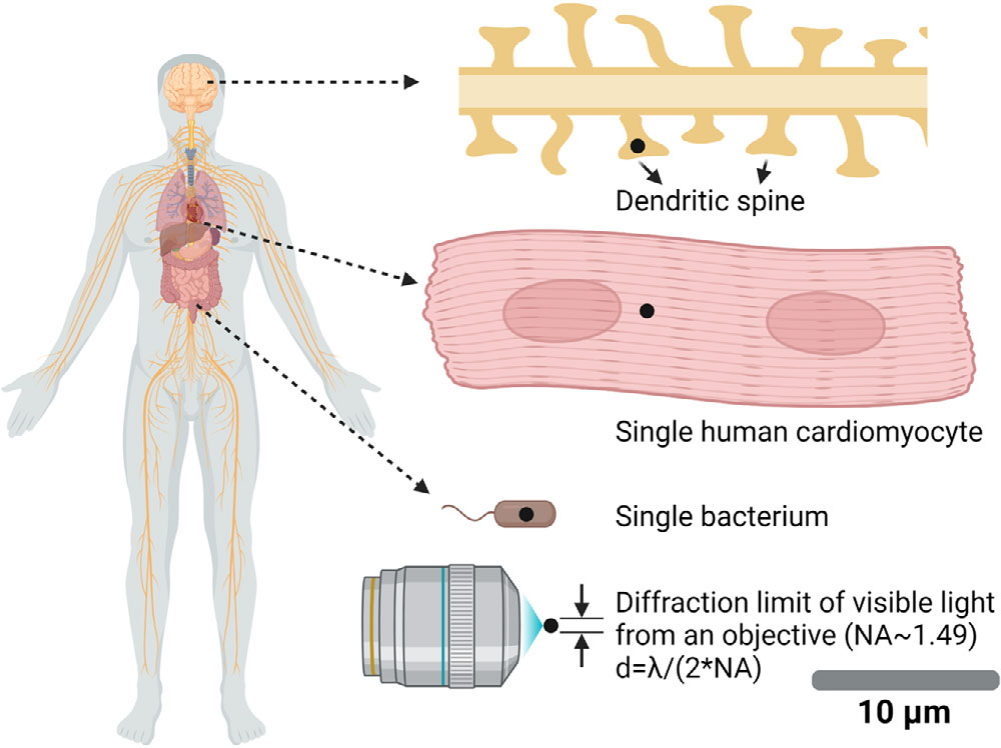
Scale of typical human cell structure and the diffraction limit of visible light. The far‐field diffraction limit of visible light (400–700 nm) is approximately half of the wavelength, significantly smaller than the typical mammalian cells such as neurons and cardiomyocytes. However, when delivered by fiber optics, scattering of light can reduce the spatial resolution but still provide sufficient spatiotemporal resolution.

Optogenetics uses light (photons) to modulate molecular activity. Compared to diffusive chemical ligands, photons can be spatially distributed in a user‐defined manner. A simple optical objective can spatially focus a coherent photon flux (e.g., a laser beam) to span about only half wavelength of the photon, reaching the far‐field diffraction limit. Fortunately, the wavelength of visible light (400–700 nm) is significantly smaller than the typical size of a cell, ranging from one micron for bacteria and tens of microns for mammalian cells (Figure 3). By systematically tuning the phase distribution of photons by devices like spatial light modulators, light can almost be distributed into any spatial pattern, for example, an array of focus, a doughnut‐shaped (as in stimulated emission depletion microscopy or STED), sheet‐shaped (as in light‐sheet microscopy), defined by the user. Note that non‐coherent light sources, such as those from a light bulb or LED, could not reach the diffraction limit because each photon has different propagation directions and phases.

### 4.2 Target specificity

Target specificity arises from the capacity to express the optogenetic protein in a tissue‐ and cell‐type‐specific manner. Recent work demonstrated the use of optogenetics in treating defective lower urinary tract (LUT) with a mouse model.^40^ LUT's physiological role, storing and emptying urine, requires coordinated, counter‐acting mechanisms of the detrusor and urethral sphincter muscles surrounding the bladder. Urination (emptying) involves the contraction detrusor and relaxation of the urethral sphincter muscles, whereas storing urine requires the opposite action from both types of muscles. However, pharmacological treatments (e.g., anticholinergics, adrenergic receptor agonists or botulinum toxin) or electronic nerve stimulation typically target upstream neurocircuits instead of muscle functions. These strategies have intrinsic drawbacks because neurocircuits regulating storing and voiding interact with other neural functions. Treatment could lead to side effects such as unwanted bowel movements or sexual functions. However, optogenetics could precisely express excitatory or inhibitory opsin proteins in the bladder smooth muscles and contract these muscles to empty urine by light. Similarly, the inhibitory opsin variant, such as HR, suppresses overactive balder symptoms, a frequent urinary bladder contraction regardless of voiding.

## 5 SELECTIVE CASE STUDIES OF TRANSLATIONAL APPLICATIONS OPSIN‐FREE OPTOGENETICS

### 5.1 Optical control of chimeric antigen receptor

Chimeric antigen receptors (CARs) are engineered recombinant receptors with an antibody‐derived ectodomain that recognizes cancer‐specific surface protein and intracellular signaling endodomains, including T‐cell activation signal CD3ζ and costimulatory molecules for activating T cells or natural killer cells.^41^, ^42^ The United State Food and Drug Administration (FDA)‐approved CAR‐T therapy targeting B cell marker CD19 has successfully treated B‐cell‐related leukemia and lymphoma.^43^ To increase the effectiveness of CAR‐T therapy on solid tumors, the new generation of CARs called ‘TRUCKs’ incorporates inducible IL‐12 production to enhance T cell activation, modulate the tumor microenvironment and recruit other immune cells.^44^ However, CAR‐T and TRUCK‐T therapy face challenges in ‘on‐target off‐tumor' toxicity and cytokine‐associated toxicity, leading to severe tissue damage and cytokine release syndrome with clinical symptoms such as nausea, fever, hypotension, vascular leakage, and life‐threatening multiple organ failure.^43^, ^44^ Anti‐inflammatory drugs and corticosteroids are used to manage the side effects of CAR‐T therapies^45^; nevertheless, adverse effects from high‐dose corticosteroids and the cost of intensive care unit treatment needed for patients with severe symptoms or high disease burden urge better strategies for increasing safety without affecting efficacy.

**FIGURE 4:**
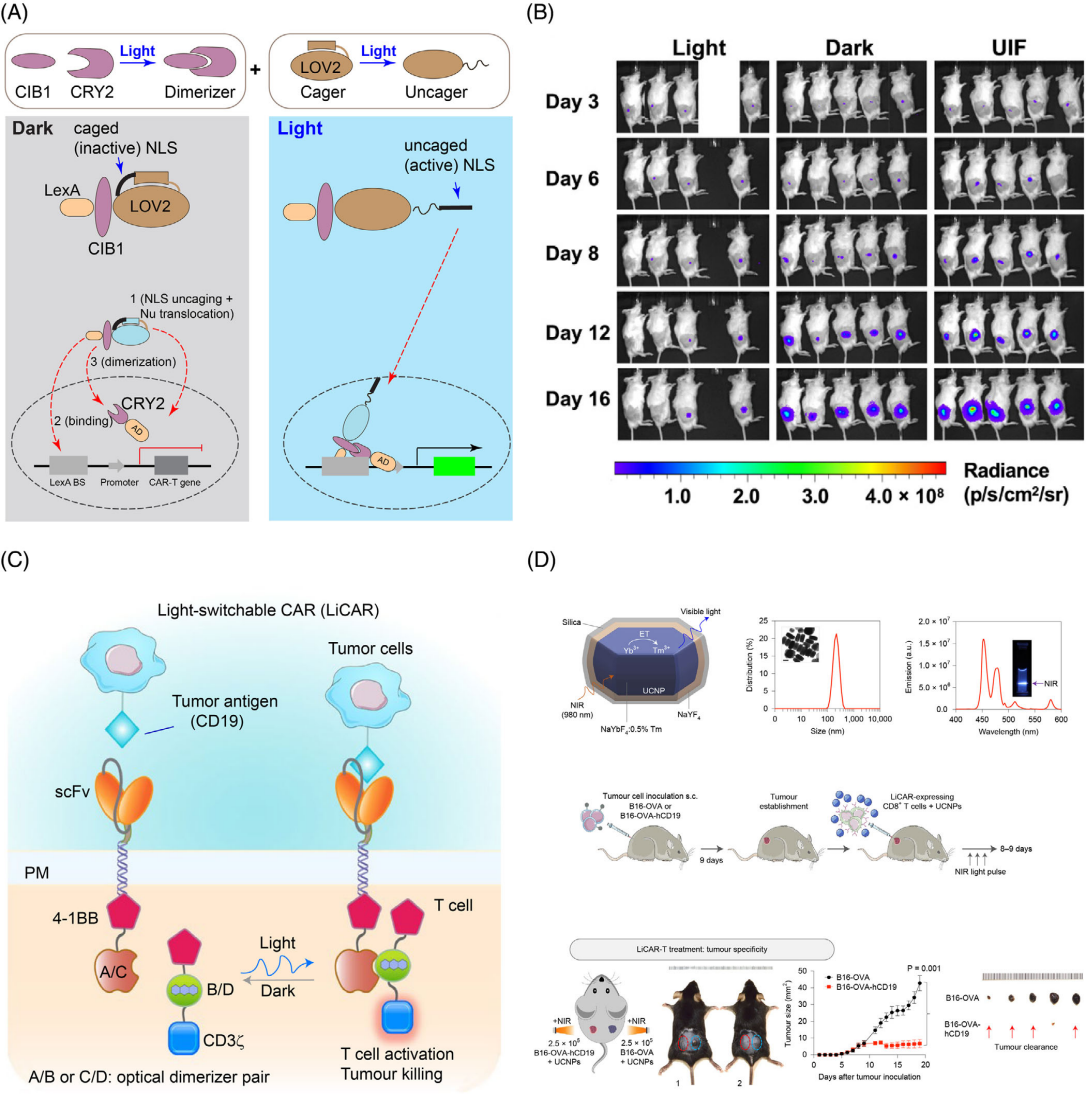
Optogenetic control of CAR. (A) CAR can be activated through light‐induced nuclear translocation of the activation domain (AD), followed by up‐regulated CAR expression. (B) Treatment of tumour cells in mice between injected with engineered T cells under light and dark treatment. (C) Light‐switchable CAR (LiCAR) recombines split CAR through light‐mediated dimerization of protein pair. T‐cell activation only occurs when reconstituted CAR recognizes tumour‐specific antigens (e.g., CD19). By injecting upconversion nanoparticles with engineered T cells, protein recombination can be achieved through near‐infrared light, which has a deeper penetration depth in biological tissues. (D) Scheme of upconversion nanoparticle and its size and spectrum distribution (top). Schematic of the experimental workflow (middle) and treatment of tumour cells with LiCAR in mice (bottom). Panel (A) and (B) are adopted from reference^46^ with permission. Panels (C) and (D) are adopted from reference^47^ with permission.

Optogenetics can increase tissue specificity and alleviate the side effects of CAR‐T therapies by providing spatiotemporal modulation. To date, two modes of action enable optogenetic control of CAR—optical induction of CAR expression or recombination of split CAR. The first strategy controls the expression of CAR by the light‐inducible gene expression system LINTAD.^46^ (Figure 4A,B) In this system, CRY2 is fused to the transcription activator VP64‐p65‐Rta (VPR) and kept in the nucleus by nuclear localization signals (NLS). CIB1 is fused to the deoxyribonucleic acid (DNA) binding domain LexA and *As*LOV2‐caged NLS, which stays cytosolic in the dark. Caging help lower the background expression as the basal level CRY2‐CIB1 pair is high.^46^ The expression of anti‐CD19 CAR was tested in mice inoculated with tumor cells Nalm‐6 and showed effectiveness in limiting the tumor size 21 days after 12‐h blue‐light illumination.^46^ LINTAD system can achieve transient expression of CAR; for sustained expression of CAR, the system relies on the Cre‐*LoxP* system and degradation of the expressed protein to achieve the temporal control of the expression.^46^

The second strategy, for example, the light‐switchable CAR (LiCAR),^47^ uses light to reconstitute split CAR protein between the costimulatory molecule 4‐1BB and activation signal CD3ζ through blue light‐sensitive CRY2‐CIBN or AsLOV2‐based iLID^48^ dimerizer system (Figure 4C,D). Combining with upconversion nanoplates, LiCAR reduced the tumour weight in mice with lymphoma after 14‐day treatment with near‐infrared (NIR) light.^47^ By modulating the activity with light, LiCAR mitigated the cytokine release syndrome and alleviated ‘on‐target off‐tumour’ effects as the level of IL‐6 is lower and the number of B cells higher in mice treated with LiCAR than those treated with traditional CAR‐T.^47^ By replacing AsLOV2 in the iLID system with circularly permutated LOV2 (cpLOV2), He and coworkers constructed cp‐iLID. They demonstrated its applications in CAR activation by blue‐light‐inducible heterodimerization of split CAR.^49^ Using a similar design, O'Donoghue and coworkers developed an optoCAR system that allows for precise modulation of the periodic activation of CAR. Intriguingly, expression of the T cell‐activation marker CD69 was attenuated with a period of 25 min of light activation (20% duty cycle).^50^ Light activation with a period shorter or longer than 25 min, even with the same integrated light input, resulted in higher CD69 expression, indicating that T cells temporally filter time‐varying signals and the CD69 expression is gated through a band‐stop filter.

Both strategies offered insights into designing light‐inducible CAR and can be expanded by other optogenetic tools. In the split‐CAR strategy, the CRY2‐CIB1‐based system showed less background activity and induced activity than the *As*LOV2‐based system,^47^ suggesting the choice of optogenetic tool may change the system's efficacy. In addition to splitting at endodomain, photoactivable removal of inhibition at ectodomain is also suggested.^51^ Basal activity should be considered in selecting activation approaches. For example, CAR expression with photoactivable Cre‐*loxP* system (TamPA‐Cre),^52^ drug‐dependent nuclear translocation was selected instead of *As*LOV2‐caged NLS.

### 5.2 Optical control of genome editing and gene expression

The Clustered Regularly Interspaced Short Palindromic Repeats or CRISPR‐Cas systems correct genomic defects by precise modification of genomic sequence. The system can also be repurposed for transcriptional regulation by using a deactivated or ‘dead’ version of Cas9 nuclease (dCas9). Like optogenetic CAR, optogenetic control of CRISPR‐Cas can be accomplished through distinct modes of action, including light‐inducible recombination of split‐Cas protein,^53^, ^54^, ^55^, ^56^, ^57^ Cas expression,^58^, ^59^, ^60^, ^61^, ^62^ and photo‐uncaging.^63^, ^64^

#### 5.2.1 Optical recombination of split‐Cas

**FIGURE 5:**
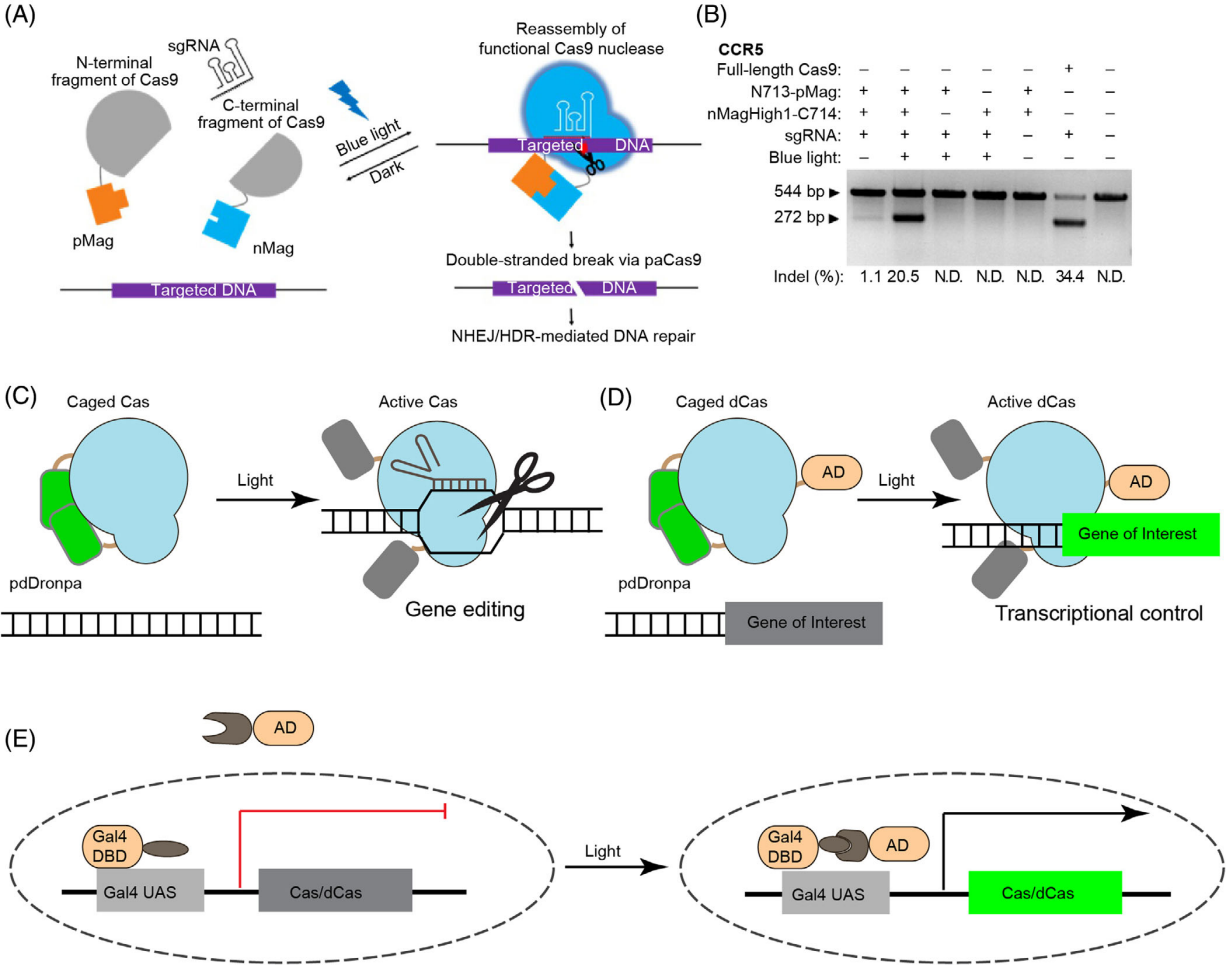
Optogenetic control of CRISPR‐Cas. (A) Optogenetic recombination of split Cas9 through blue‐light sensitive pMag/nMag protein pair and its performance in cleaving the endogenous CCR5 gene in mammalian cells (B). (C and D) Cas9/dCas9 activity can be controlled through photo‐uncaging of pdDronpa1. (E) Cas9/dCas9 expression can be controlled through optogenetic induction of gene expression. Panel (A) is adopted from reference^57^ with permission.

The first demonstration of optogenetic control of CRISPR‐Cas is the light‐mediated recombination of split‐Cas (Figure 5A,B). Both CRY2/CIB1 and VVD/Magnets pairs have been tested for fusion to N‐ and C‐SpCas9, but only the Magnets‐based split‐Cas9 (paCas9) achieved decent indel mutations (60% efficiency of the full‐length Cas9) and sequence insertion through homology‐directed repair, indicating the relative orientation between photoactivatable protein and split Cas9 determines the efficacy of recombination.^57^ The same group also developed light‐inducible CRISPR‐Cas12a (also known as CRISPR‐Cpf1 from *Lachnospiraceae bacterium*), called paCpf1, by split Cpf1 between residues 730 and 731.^54^

#### 5.2.2 Photo‐uncaging of Cas protein

Cas activity can also be controlled via photo‐uncaging.^63^, ^64^ *Rs*LOV^65^ and pdDronpa^66^ homodimerize in the dark, which can be engineered to cage Cas9 until light‐mediated protein dissociation rescues Cas9 activity. In the engineering of *Rs*LOV2‐Cas9 (paRC9), a single copy of *Rs*LOV is inserted into *Streptococcus pyogenes* Cas9 (SpCas9) between F478 and E479, and caging is achieved through dimerization of two copies of paRC9.^63^ Whereas paRC9 managed to show light‐switchability for plasmid DNA cleavage in *Escherichia coli*., careful characterization suggests that paRC9 is a ‘phenotypic’ switch that shows switchable activity in the cells (likely due to protein accumulation) but not in vitro with purified paRC9 and DNA.

The second system inserts two pdDronpa1 into SpCas9 after A259 and K1246, producing ps‐(d)SpCas9 to block the DNA binding cleft.^64^ The same approach can be applied to other Cas9 species, as the group also designed ps‐SaCas9 for *Staphylococcus aureus* Cas9 (SaCas9), with two pdDronpa1 inserted after residues 128 and 614, respectively.^64^ The ps‐SpCas9 exhibited comparable efficiency to other inducible Cas9 systems. A bonus is that pdDronpa1, a fluorescent protein variant, also indicates ps‐SpCas9 expression level through its fluorescence intensity in the cells.^64^ As exemplified by the above two cases, single‐component caging‐like control of Cas enzymes can serve both genome editing and transcriptional regulation (Figure 5C,D). The single‐component design will spare the optimization for the ratios of multiple‐component systems, and the strategy can be generalized to control other Cas enzymes for broader applications.

#### 5.2.3 Optical control of Cas protein expression

An alternative strategy is to use light to control Cas protein expression. The far‐red light (FRL)‐inducible CRISPR‐Cas12a (FICA) system^60^ and red/FRL‐mediated and miniaturized Δphytochrome A (ΔPhyA)‐based photoswitch (REDMAP)^62^ use the heterodimerization between red‐light responsive protein PhyA and its binding partner FHY1 to recruit transcription factor VP64 to the promoter and activate gene expression. A similar system based on blue‐light‐sensitive Vivid (VVD) has also been constructed^58^ (Figure 5E).

#### 5.2.4 Optical control of CRISPR‐Cas activity by modulating Cas9 inhibitors

Phage‐derived anti‐CRISPR (Acr) proteins were recently discovered as natural inhibitors of type II CRISPR systems. For example, AcrIIA4 inhibits *Streptococcus pyogenes* Cas9 through tight binding (with sub‐nanomolar affinity) to the Cas9‐sgRNA complexes. By inserting AsLOV2 into the loop L5 of AcrIIA4, Bubeck and coworkers developed CRISPR–Cas9 activity switching via a novel optogenetic variant of AcrIIA4, or CASANOVA, that allows for optical modulation of CRISPR‐Cas9 activity. In the dark, CASANOVA maintains AcrIIA4's binding to Cas9, therefore inhibits its activity; blue light stimulation causes a conformational change of AcrIIA4 (by AsLOV2) and releases Cas9 to restore its gene‐editing function.^67^ A similar strategy with anti‐CRIPSR protein AcrIIC3 also works for *Neisseria meningitidis* Cas9 (*Nem*Cas9), a smaller Cas9 with higher specificity than SpCas9.^68^

### 5.3 Optical control of gene expression

#### 5.3.1 Optical production of insulin

Light‐inducible insulin production has been demonstrated by FRL‐activated human islet‐like designer (FAID) cells in type 1 diabetes (T1D) mouse model.^69^ FAID cells are telomerase‐immortalized human mesenchymal stem cells whose genome is integrated with the BphS‐based optogenetic gene expression system to control gene expression. Selected FAID cells are implanted into the streptozotocin‐induced T1D mouse model and managed to lower the blood glucose level in a light‐dependent manner.^60^ An attractive accessory device is a self‐powered system^70^ in combination with a smartphone for remote control,^71^ which could provide alternative intervention to reduce the cost and potential side effects from the frequent injection.

#### 5.3.2 Optical regulation of cytokine release

Cancer immunotherapies suffer from severe cytotoxicity and off‐target effects. For example, bispecific T cell engagers (BiTEs), a type of bispecific antibodies that can bridge the tumor cells and T cells, elicit severe cytokine release syndrome (CRS) and neurotoxicity by uncontrolled secretion.^72^, ^73^ Hence, a far‐red‐light‐inducible expression system based on BphS modulates the toxicities by transcriptional regulation.^74^ With the optogenetic control, the cytotoxicity and cytokine release are under control by FRL illumination without losing the anti‐tumor activity.^74^ Notably, spatiotemporal control of localized T‐cell activation through recombination of split CAR also mitigates the CRS and on‐target, off‐tumor side effects.^47^

### 5.4 Optical control of mitochondrial dynamics

**FIGURE 6:**
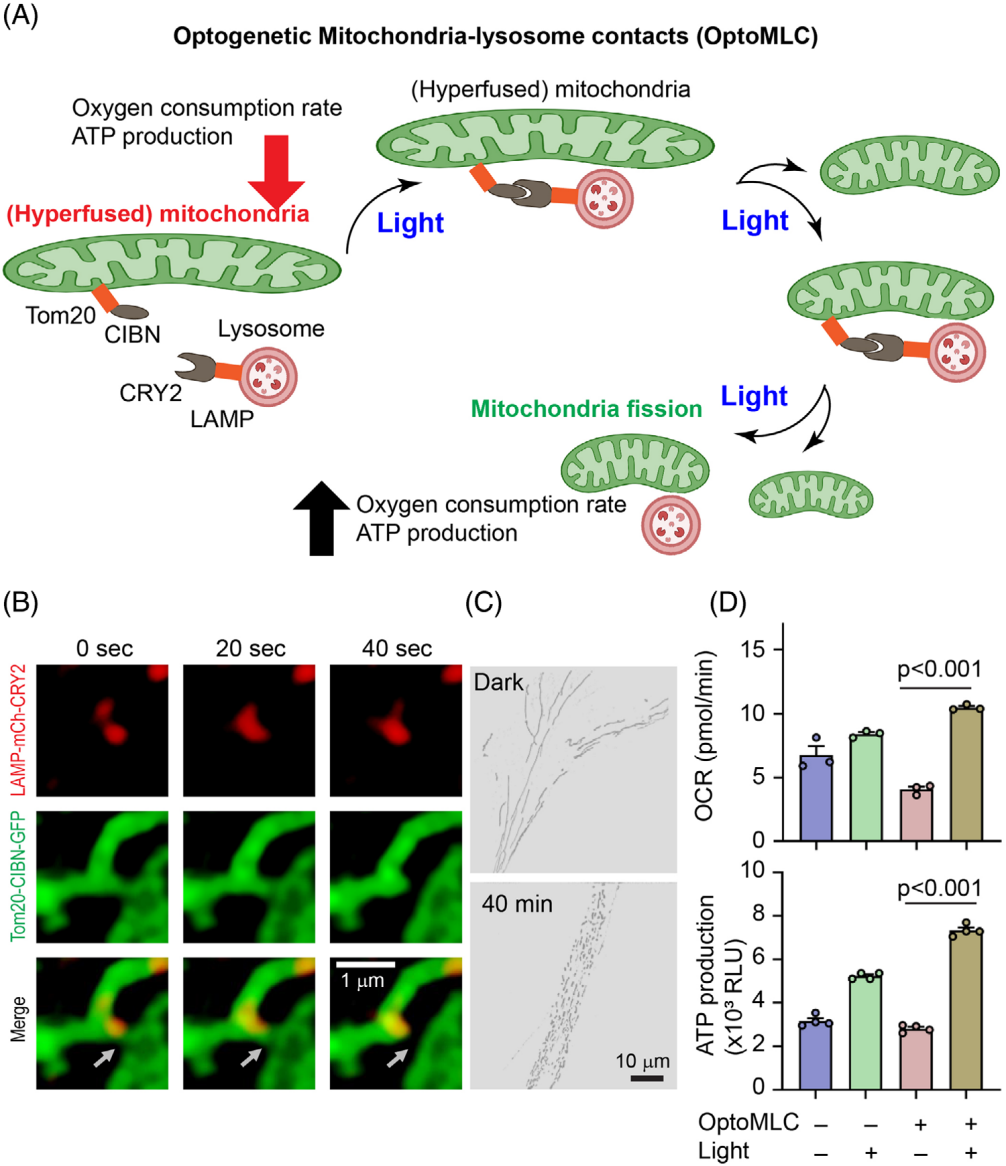
Optogenetic control of mitochondria‐lysosome contacts and mitochondrial fission. (A) Optogenetic recruitment of lysosomes to hyperfused mitochondria mediates mitochondrial fission and increases oxygen consumption rate and ATP production. (B) Time‐stamped images of the lysosome (red) and mitochondria (green) show mitochondria fission at the contact site. (C) Mitochondria morphology resolved by structural imaging microscopy before and after 40 min of blue light stimulation. More granule‐like mitochondria show after the light stimulation compared to the initial state of hyperfused mitochondria. (D) OptoMLC rescues mitochondrial function in oxygen consumption rate (OCR) and ATP production. Panel (B) to (D) are adopted from reference^77^ with permission.

Mitochondria are membrane‐bound organelles generating the majority of chemical energy through adenosine triphosphate (ATP) production. Mitochondria are also mobile organelles that exist in dynamic networks. Mitochondrial diseases are one of the most common types of inherited metabolic disorders, which affect one in 200 individuals, can present at any age, and affect any organ. Although genetic mutations that disrupt mitochondrial gene expression appear to be the most common cause of mitochondrial diseases, disorders of mitochondrial dynamics are emerging as a mechanism of disease.^75^ Optic atrophy spectrum disorder is one such disease that often involves neuron loss in different tissues. A whole‐exome sequence experiment revealed that such diseases involve mutation of SLC25A46, a gene that encodes a mitochondrial outer membrane protein. At the cellular level, a significant phenotype is the elonged hyperfused mitochondria, which show a significantly lower oxygen consumption rate and ATP production capacity. Inspired by the finding that lysosomes can regulate mitochondrial fission,^76^ Qiu and coworkers developed optoMLC (mitochondria‐lysosome contacts) that allows light‐mediated recruitment of lysosomes to mitochondria and enhanced mitochondrial fission. After light stimulation, hyperfused mitochondria in SLC25A46‐/‐ cells were significantly reduced, enhancing oxygen consumption rate and ATP production^77^ (Figure 6).

## 6 CHALLENGES OF CURRENT OPTOGENETIC STRATEGIES AND POTENTIAL SOLUTIONS

### 6.1 Routes of transgene delivery and expression

Common to all gene therapies, optogenetics‐based therapy requires the safe delivery and expression of transgenes in humans. Gene therapy has typically used viral vectors, such as adenoviruses, AAV, and lentivirus, to deliver therapeutic genes into patient tissues for long‐term expression. Each type of vector has unique strengths and associated safety concerns. Adenovirus is significantly limited in human clinical trials because its poor stable expression span and high immunogenicity. The AAV would be a better choice with more stable expression and low immunogenicity.^78^, ^79^, ^80^, ^81^

To deliver cargo to the desired tissue, gene therapists use tissue‐specific promoters, a strategy applied to target muscle cells,^82^ tumour environments in colorectal cancer^83^ and prostate cancer,^84^ and neurons.^85^ Such promoters demonstrate cell‐specific expression and therapeutic potential, and they allow higher dosing than would be possible with more widely expressed transgenes. The promoter length must be considered for this technique since many delivery methods have relatively low size limits (∼5 kbp for adeno‐associated virus).^86^

The primary safety concerns when using these vectors are the risk of viral replication within the patient, viral integration into the patient genome (or integration at undesirable loci), and the patient's immune response, as also reviewed elsewhere.^86^ Optogenetics‐based therapies are expected to introduce foreign proteins into the patient's tissue, which might exacerbate the immunological response to viral transduction. Indeed, Maimon and coworkers recently reported that AAV‐based ChR2 expression in the peripheral nervous system (PNS) requires co‐administering immunosuppressants to prevent neural death.^87^

### 6.2 Light source and delivery

Optogenetic proteins are sensitive to wavelengths of light from the near ultraviolet (UV) to the NIR. The required light wavelength and power depend on the specific protein(s) used. A combined set of light sources capable of blue (470 nm), orange (560 nm), and infrared (>700 nm) can activate the majority of currently available optogenetic proteins.^88^ Light delivery poses a challenge concerning power requirement and correct positioning. With ample surface areas of tissues like muscle or the brain to be illuminated, demands of powering light sources become a limiting factor. The primary sources of light delivery are optic fibers and optical implants. While wireless optic sources offer opportunities for specific targeting and precise control, the power limit of these devices reduces the operational limit to 3 cm, a depth to be further optimized for human operation.^89^

Most hospitals are already equipped with medical lasers and advanced optics for techniques such as photodynamic therapy.^90^, ^91^ Many of these light sources are much higher power than necessary for optogenetic therapies and can be easily filtered and coupled to fiber optic cables for tissue delivery. However, new devices must be manufactured and approved when surgical intervention is required. Clinical approval of these devices is a lengthy process, and establishing which regulations apply will require communication with regulatory bodies, especially if clinical trials recruit participants from multiple countries. The safety of surgically implanted devices must be carefully considered. Devices designed to illuminate internal organs with light must operate without generating excess heat, and implanted devices could complicate specific diagnostic techniques such as magnetic resonance imaging (MRI).^92^

Improvement in light delivery systems can significantly improve the efficacy of optogenetic tools. A recent study focused on using a time‐reversed ultrasonically encoded (TRUE)‐based light delivery system to accurately focus light and avoid light scattering of the light 89. The TRUE technique could control neural firing rate much more efficiently than conventional light focusing techniques. However, the TRUE system can be ineffective if the system response time exceeds the decorrelation time of the tissue of interest. For example, decorrelation time can drop due to blood flow and muscle movements. Besides, the weight and size of light‐delivering implants could significantly impede a patient's daily activities.

### 6.3 Photo and thermal toxicity from light stimulation

Senova and coworkers^93^ studied the spatial distribution of light and its effects in large brain volumes of animal models. This study demonstrates that deep light penetration in the brain cortex is possible without detrimental photo and thermal damage to the organ. The authors reported that 1990 seconds of light administration with an irradiance of 100 to 600 mW/mm^2^ and 5 ms pulse duration at 20, 40, and 60 Hz did not trigger non‐physiological functional activation. Optimization of stimulation parameters is necessary to avoid potential damage to deep tissues. Assessment of phototoxicity of long‐term photo‐stimulation requires further investigation.

### 6.4 Bioavailability

Some photoactivatable proteins require exogenous cofactors, whose bioavailability becomes an essential measure for assessing the efficacy of any light‐based therapy. Exogenous cofactors like plant‐derived PCB are required for light absorption into various red‐shifted photoactivatable proteins such as PhyB. PCB supply must be ensured via transgenic co‐expression of PCB biosynthesis genes or by an external supply of synthetic PCB. Various PCB‐providing dietary supplements like blue‐green algae spirulina could be administered orally. However, the supplement dosage requirement for effective phytochrome activation remains to be validated.^94^ Improved optogenetic tools should minimally depend on external cofactors or use endogenous cofactors in mammals such as biliverdin, FAD, FMN or riboflavin.

## 7 CONCLUSIONS AND PERSPECTIVE

Light has been used clinically to treat or mitigate diseases such as insomnia, depression,^95^ and cancer.^96^ For sleeping disorders, the physiological foundation of light treatment is that living organisms develop biological rhythms at approximately a 24‐hr cycle. Patterned light treatment synchronizes the physiological function, for example, the brain's regulation of melatonin and temperature, to the 24 h, so patients are asleep at night and awake during the day. The mechanisms by which light affects mood are less understood, and circadian rhythms are believed to be involved. Photodynamic therapy treats cancers by using light and photosensitizers to generate reactive oxygen species that can damage cancer cells. These therapies, however, primarily use the radiation of light waves and do not provide spatiotemporal accuracy and target specificity.

Emerging optogenetics addresses these challenges by genetically targeting cells with specificity and spatiotemporal control of molecular activity. Current clinical trials of optogenetics focus on treating vision loss because the retina has long been considered the ‘approachable’ part of the brain. Therefore, researchers and entrepreneurs have capitalized on the accessibility of the retina to investigate essential translational applications of optogenetics. However, as showcased in this work, optogenetics is blessed with significant flexibility in its modality to control molecular activity, cell signaling, and physiological functions. Although issues concerning light delivery, transgene delivery, and phototoxicity remain, we expect that continuous technical advancements, such as in nanoscience and engineering, will eventually overcome these challenges and bring optogenetics into the healthcare sector as a powerful precision medicine tool. We also hope the field can embrace discussion and dialog regarding ethical perspectives, an issue faced by many relevant fields, such as cell and gene therapy, in translational research.

## CONFLICT OF INTEREST STATEMENT

The authors declare no potential conflict of interest.
